# Salt-responsive lytic polysaccharide monooxygenases from the mangrove fungus *Pestalotiopsis* sp. NCi6

**DOI:** 10.1186/s13068-016-0520-3

**Published:** 2016-05-20

**Authors:** Ilabahen Patel, Daniel Kracher, Su Ma, Sona Garajova, Mireille Haon, Craig B. Faulds, Jean-Guy Berrin, Roland Ludwig, Eric Record

**Affiliations:** INRA, UMR1163 Biodiversité et Biotechnologie Fongiques, Aix-Marseille Université, Polytech Marseille, 163 Avenue de Luminy, CP 925, 13288 Marseille Cedex 09, France; UMR1163 Biodiversité et Biotechnologie Fongiques, Faculté des Sciences de Luminy-Polytech Marseille, Aix-Marseille Université, 163 Avenue de Luminy, CP 925, 13288 Marseille Cedex 09, France; Department of Food Sciences and Technology, Food Biotechnology Laboratory, BOKU-University of Natural Resources and Life Sciences, Muthgasse 18, Vienna, 1190 Austria

**Keywords:** AA9, LPMO, Cellobiose dehydrogenase, Cellulose, Biorefinery

## Abstract

**Background:**

Lytic polysaccharide monooxygenases (LPMOs) belong to the “auxiliary activities (AA)” enzyme class of the CAZy database. They are known to strongly improve the saccharification process and boost soluble sugar yields from lignocellulosic biomass, which is a key step in the efficient production of sustainable economic biofuels. To date, most LPMOs have been characterized from terrestrial fungi, but novel fungal LPMOs isolated from more extreme environments such as an estuary mangrove ecosystem could offer enzymes with unique properties in terms of salt tolerance and higher stability under harsh condition.

**Results:**

Two LPMOs secreted by the mangrove-associated fungus *Pestalotiopsis* sp. NCi6 (*Ps*LPMOA and *Ps*LPMOB) were expressed in the yeast *Pichia pastoris* and produced in a bioreactor with >85 mg L^−1^ for *Ps*LPMOA and >260 mg L^−1^ for *Ps*LPMOB. Structure-guided homology modeling of the *Ps*LPMOs showed a high abundance of negative surface charges, enabling enhanced protein stability and activity in the presence of sea salt. Both *Ps*LPMOs were activated by a cellobiose dehydrogenase (CDH) from *Neurospora crassa*, with an apparent optimum of interaction at pH 5.5. Investigation into their regioselective mode of action revealed that *Ps*LPMOA released C1- and C4-oxidized cello-oligosaccharide products, while *Ps*LPMOB released only C4-oxidized products. *Ps*LPMOA was found to cleave polymeric cellulose in the presence of up to 6 % sea salt, which emphasizes the use of sea water in the industrial saccharification process with improved ecological footprints.

**Conclusions:**

Two new LPMOs from the mangrove fungus *Pestalotiopsis* sp. NCi6 were found to be fully reactive against cellulose. The combined hydrolytic activities of these salt-responsive LPMOs could therefore facilitate the saccharification process using sea water as a reaction medium for large-scale biorefineries.

**Electronic supplementary material:**

The online version of this article (doi:10.1186/s13068-016-0520-3) contains supplementary material, which is available to authorized users.

## Background

Pessimistic forecasts of fossil-fuel reserves and the environmental impacts of oil production and use have spurred extensive worldwide research into the conversion of different local lignocellulosic biomasses as alternative sustainable sources for biofuel production [1–4]. Lignocellulosic plant cell walls are very resistant to degradation, as cellulose and hemicellulose are embedded into a lignin matrix which limits the ability of cellulases, the major hydrolytic enzymes in the saccharification process, to access their sites of action. The term “cellulases” covers a cocktail of enzymes that are exploited for the deconstruction or modification of plant biomass within the biorefinery concept [4–6]. However, cost-effective use of such enzyme cocktails is a limiting step for the production of biofuels and commodity chemicals, making the discovery of efficient and more robust enzymes from biodiversity a necessary challenge.

Copper-dependent lytic polysaccharide monooxygenases (LPMOs) are emerging as important components of the cellulase cocktail, [7–9], where they have even been described as “cellulase boosters” [10]. Recent intensive efforts have started to unravel their function in the oxidative degradation of cellulose [11–14] and other plant polysaccharides such as hemicellulose [9] and starch [15, 16]. LPMOs are currently classified as “auxiliary activities (AA)” within 4 families of the Carbohydrate Active Enzyme (CAZy) database [17, 18]: the fungal AA9, formerly known as glycosyl hydrolases 61; the bacterial AA10, formerly known as carbohydrate-binding modules 33 [17]; the newly characterized AA11 [19]; and the starch-oxidative LPMOs of AA13 [16]. Although the CAZy database contains more than 200 AA9 proteins, less than 20 have been enzymatically characterized and shown to oxidatively cleave the β-(1→4) glycosidic bonds of cellulose [20, 21]. AA9 LPMOs share a common structural fold with a flat substrate-binding surface, a conserved N-terminal histidine residue involved in the coordination of an essential copper ion, and the dependency of the activity on the presence of an electron donor. The reaction mechanism of the majority of the characterized LPMOs involves oxidation of the C1 carbon of a glucose molecule, leading to the formation of an aldonic acid and a break in the cellulose chain, whereas other members of the AA9 family have been shown to generate oxidation at C4 [20, 22]. AA9 LPMOs can be split into three classes on the basis of preferred site of oxidation, i.e., type 1 oxidizing at C1, type 2 oxidizing at C4, and type 3 oxidizing at both the C1 and C4 carbon atoms of glucose [15]. The oxidative cleavage performed by LPMOs occurs in the presence of small redox-active molecules such as ascorbic acid, reduced glutathione, or gallate [8, 11, 23]. The peculiarity of fungal AA9 LPMOs is their action in concert with cellobiose dehydrogenases (CDHs) that results in redox-mediated glycosidic bond cleavage in cellulose and points to a key role of this oxidative system in fungi [21, 24, 25].

Many LPMOs have been successfully produced heterologously from fungi, but few reports have detailed their biochemical characterization. One reason is the lack of a fast direct activity test. Kittl et al. [26] found that the Amplex Red assay can be modeled for biochemical characterization of LPMOs based on the generation of hydrogen peroxide as a side-reaction of the enzyme which is dependent on the availability of a suitable reductant for the LPMO type-2 copper center.

For the sustainable success of emerging biorefineries, apart from the need of novel biocatalytic systems another important aspect to consider is the large water consumption that lignocellulose biomass-processing plants will require at large-scale operations [27]. The assessment of non-potable water resources as reaction media for such biorefineries appears to be a promising field of research, which can result in integrative systems [28]. From these viewpoints, novel bio-catalysts should demonstrate its reactivity in crude aqueous media such as seawater. The marine fungi genera *Pestalotiopsis* sp. have lignocellulolytic enzymes of interest in terms of high salt tolerance and higher stability under harsh conditions [29]. Hence, we selected two putative *lpmo* genes from *Pestalotiopsis* sp. NCi6 (*Ps*) [30] for heterologous expression in the yeast *Pichia pastoris.* Their regioselectivity was tested on cellulose in the presence and the absence of sea salt for the possible application in the degradative processes of plant cell wall in saline/non-saline environment.

## Results and discussion

### Gene expression and batch-scale production of LPMOs

**Fig. 1:**
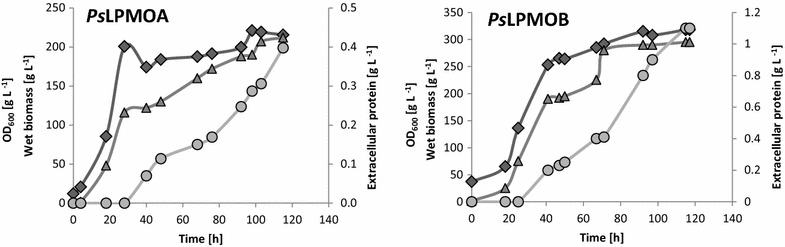
Production of *Ps*LPMOs in *P. pastoris*. *Squares* wet biomass; *triangles* optical density (OD); *circles* extracellular protein concentration

Our previous transcriptome analysis of the mangrove fungus *Pestalotiopsis* sp. NCi6 identified five putative *lpmo* sequences, two of which were strictly secreted under saline growth conditions. Three other candidates, one having a CBM, were found in both saline and non-saline growth conditions [30]. Among them, *lpmoa* (only secreted in saline conditions) and *lpmob* (secreted in saline/non-saline conditions) were chosen for heterologous production in *P. pastoris*. The two genes were selected based on the following criteria: (1) the corresponding proteins were secreted by *Pestalotiopsis* sp. NCi6 when grown on mangrove wood [30]; (2) one protein was strictly induced in saline conditions (*Ps*LPMOA), while the second protein was present in both saline and non-saline growth conditions (*Ps*LPMOB) [30]; (3) the amino acid sequences are diverse among each other and compared to previously characterized LPMOs (Additional file 1: Figure S1). The selected *Ps*LPMOs have a sequence identity of only 40 % and belong to two separate phylogenetic branches, although they share a strictly conserved copper-binding site which consists of two histidines (one at N-terminal position) and one tyrosine. The codon-optimized genes encoding *Ps*LPMOA and *Ps*LPMOB were inserted into the pPICZαA expression vector for subsequent expression in the yeast *P. pastoris*. The best transformants that showed high protein secretion and the presence of masses corresponding to the expected molecular mass of *Ps*LPMOA and *Ps*LPMOB (as estimated by SDS-PAGE) were selected for production in 500-mL submerged cultures under agitation. A 2-L bioreactor was chosen for a scale-up protein production. Fermentation of the *Ps*LPMOA-producing transformant was performed with 1.5 % sea salt in the batch cultivation medium to get active enzyme, whereas *Ps*LPMOB was cultivated without sea salt. These cultivations were monitored for wet biomass, extracellular protein concentration (Fig. 1). LPMO secretion was verified by SDS-PAGE (data not shown). After the batch and glycerol fed-batch phases for biomass build-up, wet biomass concentrations in the fermentations of *Ps*LPMOA and *Ps*LPMOB reached 200 and 130 g L^−1^, respectively. Both bioreactors were harvested after around 118 h with protein levels of 0.4 and 1.0 g L^−1^ for *Ps*LPMOA and *Ps*LPMOB, respectively. These protein yields are comparable with other previously characterized LPMOs from terrestrial fungi [21, 25].

The culture supernatants were harvested and purified by single-step affinity chromatography (Table 1). *Ps*LPMOB was purified to homogeneity, whereas *Ps*LPMOA showed two bands on SDS-PAGE (Additional file 1: Figure S2), suggesting that two isoforms were co-produced. This could be a result of varying protein processings, such as a difference in glycosylation. SDS-PAGE analysis showed that both proteins are heavily glycosylated resulting in diffuse bands of considerably larger sizes than the masses expected from the amino acid sequences. Deglycosylation with PNGase F under denaturing conditions was performed, but no visible effect on the molecular mass or appearance of the proteins on SDS-PAGE was observed for *Ps*LPMOB (Additional file 1: Figure S2). This is consistent with the fact that no N-glycosylation sites were predicted for *Ps*LPMOB. Conversely, *Ps*LPMOA possesses two N-glycosylation sequences, and deglycosylation with PNGase F showed a clear shift yielding a unique band, the upper band corresponding to the PNGase F. The terminal sequence of the *Ps*LPMOA, as determined following an Edman degradation on the purified protein, corresponded to HYTFPD, indicating that this protein was correctly matured in *P. pastoris*. On the other hand, the N-terminal sequence of *Ps*LPMOB showed a mix of two sequences, EAEAHTI (60 % abundance) and HTI (40 %), revealing that the main portion of the protein was not correctly processed. The presence of the N-terminal histidine is essential because it coordinates the active-site copper ion, which is involved in oxygen activation to cleave glycosidic bonds in cellulose. An additional amino acid at the N-terminus, as was observed for *Ps*LPMOB (Glu, Ala, Glu, Ala) would most likely render the protein inactive [31]. Immunodetection of the *Ps*LPMOs was performed using antibodies raised against the poly-HIS tag. Western blotting showed bands corresponding to the mass of proteins, demonstrating that *Ps*LPMOA and *Ps*LPMOB recombinant proteins were not truncated at the C-terminus.

**Table 1:** Purification steps using immobilized metal affinity chromatography (IMAC) of recombinant LPMOs

	Purification	Volume (mL)	Total activity (U)	Protein (mg)	Specific activity (U g^−1^)	Activity yield	Purification factor (fold)
*Ps*LPMOA	Culture medium	1500	1.2	495	2.42	100	1.0
	IMAC	2.7	0.51	130	3.92	42.5	1.6
*Ps*LPMOB	Culture medium	1060	0.25	1160	0.22	100	1.0
	IMAC	1.9	0.62	285	2.18	248.0	9.9

### Structural basis of salt tolerance

**Fig. 2:**
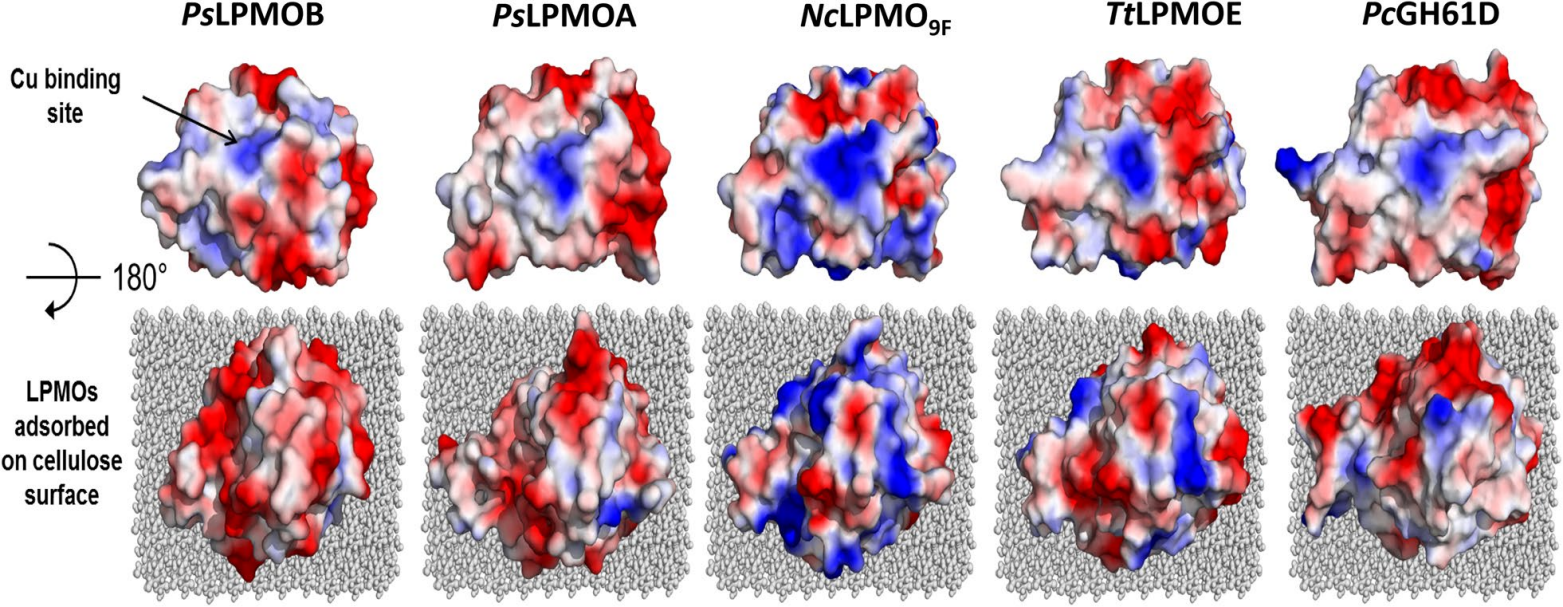
Surface charge plots of *Ps*LPMOA and *Ps*LPMOB, *N. crassa* LPMO_9F_ (UniProt Q1K4Q1 [34]), *H. jecorina* CEL61B (UniProt: Q7Z9M7 [35]), and *P. chrysosporium* GH61D (UniProt: BAL43430 [36]). *Top panels* illustrate the copper-binding “histidine brace” on the flat cellulose-binding patch (indicated by *arrows*). LPMOs adsorbed on cellulose (*gray sheets*) are shown in the *lower panels*. Surface charge distribution is shown in *color* code (*red* negative charges; *blue* positive charges). The surface potentials were calculated using the vacuum electrostatics function of the PyMOL molecular graphics system (Schrödinger, New York, NY)

Salt-adapted hydrolases typically contain a high number of negative surface charges that enable enhanced protein stability and activity in extreme osmolytic habitats [32, 33]. To test whether this feature also applies to the *Ps*LPMOs, structure-guided homology models were generated and their surface charge distribution was compared to LPMOs from other fungal origins. The overall topologies of the homology models bear the hallmarks of cellulose-active LPMOs including a central β-sandwich fold and a partially solvent-exposed active-site copper chelated by a histidine brace (Additional file 1: Figure S3). The “flat face” of LPMOs, which is oriented toward the cellulose substrate during catalysis, seems to contain a fairly balanced number of positive and negative surface charges in all investigated structures (Fig. 2). The residual solvent-exposed surfaces of *Ps*LPMOA and *Ps*LPMOB, however, showed a high abundance of negative charges relative to LPMO_9F_ from *Neurosporacrassa* [34] and *Hypocrea jecorina* Cel61B [35]. *Ps*LPMOA and *Ps*LPMOB showed a 4.8-times and 4.3-times higher abundance of negative amino acids (D + E) over positively charged amino acids (R + K). None of the 21 LPMO sequences analyzed along with the *Ps*LPMOs showed an equally high ratio of (D + E)/(R + K), although the sequences of the thermophilic fungi *Thermoascus aurantiacus* [12] (ratio 2.7) and *Myriococcum thermophilum* MYCTH112089 [34] (ratio 2.0) as well as LPMO GH61D from the mesophilic basidiomycete *Phanerochaete chrysosporium* [36] (ratio 2.4; Fig. 2) showed a significantly higher number of Asp and Glu residues than other non-halophilic LPMOs (Additional file 1: Table S1). These examples illustrate that dominance of negative surface charges is a strong indication for adaption to saline conditions, but is sometimes also observed in LPMOs from non-halophilic organisms. *Ps*LPMOs tend to have lower isoelectric points (pI) than other fungal LPMOs. The experimentally determined pIs of *Ps*LPMOA and *Ps*LPMOB were between pH 4.2 and 4.7, which is in good agreement with the calculated pIs based on the amino acid composition.

### Activity determination, pH, and temperature stabilities

**Fig. 3:**
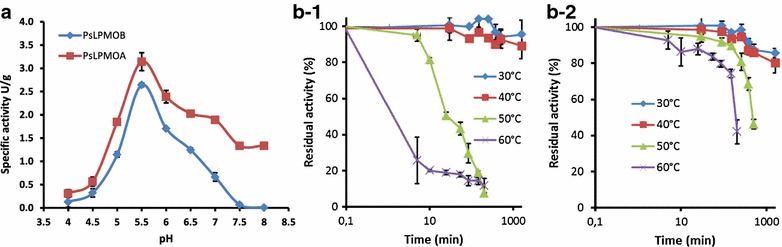
Amplex Red-based *Ps*LPMOs characterization. **a** pH profiles of *Ps*LPMOs (*red line Ps*LPMOA; *blue line* *Ps*LPMOB). **b-1** Temperature stability of *Ps*LPMOA and **b-2** temperature stability of *Ps*LPMOB (*diamonds* 30 °C; *squares* 40 °C; *triangles* 50 °C; *stars* 60 °C)

The high abundance of negative surface charges of the *Ps*LPMOs relative to other LPMOs had no apparent effect on the interaction with the CDHs from *N. crassa*. Initial characterization of the recombinant *Ps*LPMOs was carried out by measuring their capacity to produce H_2_O_2_ in the absence of a cellulosic substrate. We found that the two *Ps*LPMOs were readily reduced by both *N. crassa* CDHs (*Nc*CDHIIA, UniProt: Q7RXM0 and *Nc*CDHIIB, UniProt: Q7S0Y1), indicating productive interactions. In addition, the reducing agent ascorbic acid also gave a concentration-dependent signal with the *Ps*LPMOs. Oxygen turnover by *Ps*LPMOs was routinely measured in combination with *Nc*CDHIIA using its native substrate cellobiose. Specific oxygen-reducing activities of 3.9 and 2.2 U g^−1^ were determined for *Ps*LPMOA and *Ps*LPMOB, respectively. These values are in the same range as previously published LPMOs from terrestrial fungi [21, 26]. The lower specific activity of *Ps*LPMOB could be related to the fact that only 40 % of the enzyme is correctly processed. The effects of pH and temperature on enzyme stabilities were determined within a pH range from 4.0–8.0. The pH-dependent activities for both *Ps*LPMOs showed bell-shaped curves with optima at pH 5.5 (Fig. 3a). Both enzymes retained more than 50 % residual activity between pH 5.0 and pH 6.5. This is in good agreement with the pH-dependence of *Nc*CDHIIA for artificial one- and two-electron acceptors [24]. At more acidic pH-values below 4.5, no activity could be detected. This could be related to the low pIs of both *Ps*LPMOs. The high density of negative charges on their surfaces could result in substantial charge repulsion at the CDH/LPMO interfaces at low pH and potentially prevent effective electron exchange, as was shown for the internal cofactor-to-cofactor electron transfer of a CDH from *M. thermophilum* [34]. The effect of sea salt (3.5 % w/v) on the thermal stability of the *Ps*LPMOs was examined at 50 °C. As shown in Additional file 1: Figure S4, sea salt had no noticeable effect on *Ps*LPMOA, which showed similar half-lives under all experimental conditions, but a strong negative effect on *Ps*LPMOB, which showed half-lives shortened below 5 min. Temperature stabilities of *Ps*LPMOA and *Ps*LPMOB measured in the absence of sea salt are shown in Fig. 3b-1, b-2, respectively. Both enzymes were stable at 30 and 40 °C, retaining 100 % activity for 150 min and approximately 80 % after 1000 min of incubation. *Ps*LPMOB was considerably stable at 50 and 60 °C, whereas *Ps*LPMOA showed fast degradation at 60 °C with an estimated half-life of 2 min. At 50 °C, *Ps*LPMOA had a ten-fold shorter half-life than *Ps*LPMOB. To the best of our knowledge, pH-dependent activities and temperature-profiles of LPMOs have not been reported so far. The thermal stability of four *N. crassa* LPMOs was evaluated in a previous study using differential scanning calorimetry, which showed similar transition midpoint temperatures ranging from 63.0–68.9 °C for all enzymes [26].

### Cellulose degradation capacity and oxidative regioselectivity of LPMOs

**Fig. 4:**
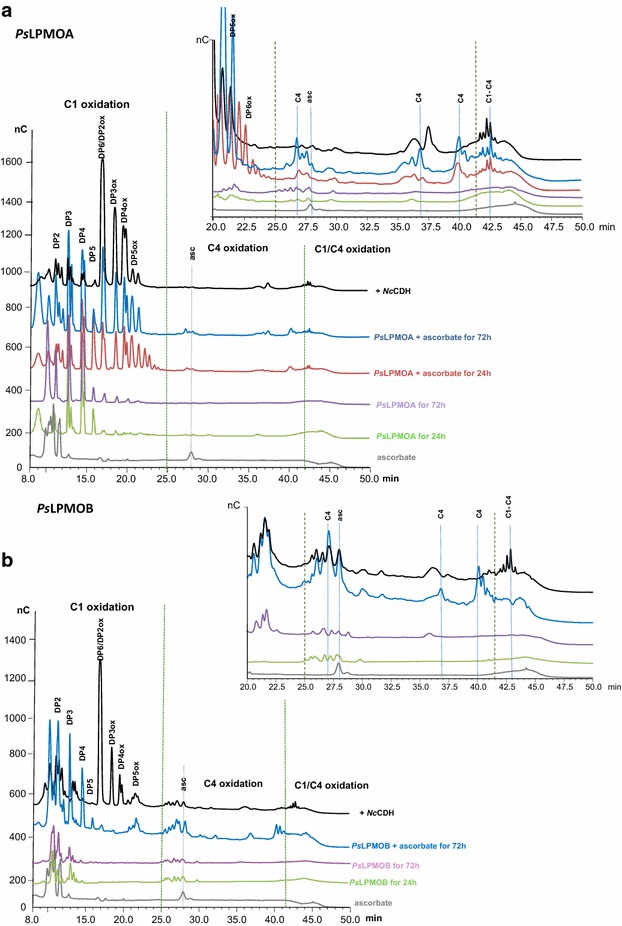
Analysis of oxidized products generated from cellulose by action of *Ps*LPMOs. **a** HPAEC chromatograms of the oligosaccharides products released by action of *Ps*LPMOA. **b** HPAEC chromatograms of the oligosaccharides products released by action of *Ps*LPMOB. The peak annotations are based on comparison with oligosaccharide standards oxidized at the C1 position (DP2ox-DP5ox). Peaks eluting at 27, 37, and 41 min are annotated with *dotted lines*. *Controls* ascorbate (*gray line*), *Ps*LPMO with PASC for 24 h (*green line*) or 72 h (*purple line*). *Reaction samples* PASC (0.1 %) with 4.4 µM *Ps*LPMOs in the presence of 1 mM ascorbate, at 50 °C for 24 h (*blue line*) or 72 h (*red line*), and same reaction followed by the incubation with *Nc*CDHIIA at 50 °C for 16 h (*black line*)

To investigate the regioselectivity of the *Ps*LPMOs, activity assays were performed on phosphoric acid swollen cellulose (PASC) in the presence of ascorbic acid as electron donor. Product profiles were resolved by high-performance anion-exchange chromatography (HPAEC) as described in previous works [21, 37]. Figure 4a shows HPAEC elution pattern of a released mixture of non-oxidized and oxidized soluble oligosaccharides for *Ps*LPMOA. The degrees of polymerization (DP) of the products ranged from DP2 to DP5 for both non-oxidized and oxidized oligosaccharides. C1-oxidized oligosaccharides with DPs from two to five were observed in the assay on *Ps*LPMOA, indicating that its action on cellulose yields C1-oxidized oligosaccharides. Generated lactones spontaneously hydrolyze to aldonic acids in bulk water. In 24-h reactions, we also observed a small peak corresponding to C1–C4 oxidation at around 42–44 min in the chromatogram for *Ps*LPMOA, while *Ps*LPMOB showed no oxidized oligosaccharide products at similar reaction time. Prolonging the reaction time from 24 to 72 h to clarify the regioselectivity of LPMOs resulted in C1-oxidized oligosaccharides and other peaks which eluted at 27, 37, and 40 min for *Ps*LPMOA (zoom panel, Fig. 4a), while only the three later peaks were observed for *Ps*LPMOB, as shown in Fig. 4b. These latter peaks correspond to the C4-oxidized species as was confirmed by Bennati-Granier et al. [21] using Mass Spectrometry analysis. The soluble fraction of the 72 h reaction from *Ps*LPMOs was further treated with *Nc*CDHIIA to oxidize the reducing (C1) ends of the C4-oxidized cello-oligosaccharides. Analysis showed a loss of peaks probably corresponding to C4-oxidized species and an increase in peaks at later retention times (Fig. 4). For both *Ps*LPMOA and *Ps*LPMOB, addition of *Nc*CDHIIA led to an increase of C1-oxidized oligosaccharides and a clear change of products eluting after 25 min. Indeed, oxidized species at 27, 37, and 40 min (C4-oxidized species) vanished with the concomitant appearance of new peak at around 42–44 min, which based on the analysis [21, 22] may correspond to a C1/C4-double-oxidized DP2 and longer double-oxidized products, respectively. In conclusion, these experiments confirm that oxidative cleavage of cellulose occurs at both C1 and C4 for *Ps*LPMOA but only at C4 for *Ps*LPMOB. Comparison with results from similar experiments [21, 22, 25] shows that the purified proteins are active LPMOs and emphasizes the importance of CDH as electron donor. It has been suggested that the presence of native cellodextrins could originate from oxidative cleavages near the reducing ends releasing an intact non-reducing moiety [7, 11]. *Ps*LPMOB produced mainly peaks corresponding to C4-oxidized cello-oligosaccharides, whereas *Ps*LPMOA produced doubly oxidized compounds corresponding to C4 oxidation (4-keto or gem-diol sugar) and C1 oxidation (aldonic acid). Furthermore, we showed that *Ps*LPMOB falls into the class of type-2 LPMOs (C4 oxidation), which fits with the regioselectivity pattern previously predicted based on phylogenetic analysis [15]. However, ionic chromatography evidenced that *Ps*LPMOA behaves as a type-3 LPMO (oxidation at the C1 and C4 positions), although it is predicted to be a type-1 LPMO (oxidation at C1 position only); suggesting that this classification based on sequence alignments may be unable to depict the full scope of action in LPMOs. It is therefore essential to note that AA9s show low overall sequence identities across the entire family [20, 21, 38].

### Cellulose degradation in the presence of sea salt

**Fig. 5:**
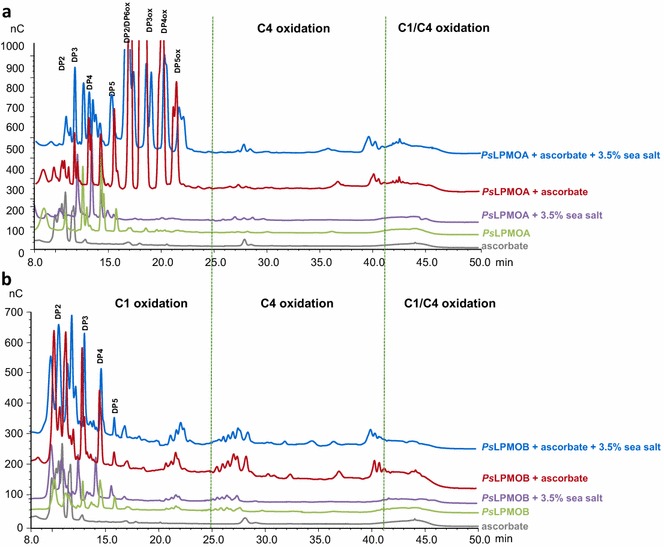
HPAEC chromatogram showing products released in the presence and absence of sea salt by action of *Ps*LPMOs. **a** Cellulose degradation products by action of *Ps*LPMOA. **b** Cellulose degradation products by action of *Ps*LPMOB. The peak annotations are based on comparison with oligosaccharide standards oxidized at the C1 position (DP2ox-DP5ox). *Controls* ascorbate (*gray line*), *Ps*LPMO with PASC without sea salt for 72 h (*green line*), or with 3.5 % sea salt (*purple line*). *Reaction samples* PASC (0.1 %) with 4.4 µM *Ps*LPMOs with 1 mM ascorbate in the presence of 3.5 % sea salt, at 50 °C for 72 h (*blue line*) and same reaction without sea salt (*red line*)

As *Ps*LPMOA was produced under saline conditions, we used a cellulose cleavage assay to test the effect of sea salt on the performance of this enzyme. Activity on PASC with ascorbate as the electron donor was measured by HPAEC in presence of different concentrations of sea salt. Figure 5 shows that both *Ps*LPMOs were fully active in the presence of up to 3.5 % (w/v) of sea salt. Both the HPAEC chromatograms showed slight shifts of oxidized product peak retention times due to the presence of sea salt in the samples. Nevertheless, significant amounts of C1- and C4-oxidized oligosaccharides were detected in the presence and the absence of sea salt for *Ps*LPMOA, whereas (only) C4-oxidized oligosaccharides were detected for *Ps*LPMOB. Furthermore, *Ps*LPMOA remained active even at 6.0 % (w/v) sea salt (Additional file 1: Figure S5), whereas *Ps*LPMOB showed no activity at this sea salt concentration. These results are contrasted by the peroxide-detecting Amplex Red assays (Additional file 1: Figure S3), which showed fast inactivation of *Ps*LPMOB in the presence of 3.5 % (w/v) sea salt (Additional file 1: Figure S3). These assays, however, were performed in the absence of a suitable substrate for LPMO, and may therefore indicate that binding of LPMO to polysaccharide surfaces contributes to the overall stability of the enzyme.

## Conclusions

*Pestalotiopsis* sp. NCi6 contains a complete arsenal of cellulolytic enzymes and represents an interesting model organism for studying the oxidative deconstruction of lignocellulose in extreme halophilic habitats. Here, we successfully produced two *Ps*LPMOs (*Ps*LPMOA and *Ps*LPMOB) and characterized them in depth. The Amplex Red assay was found to be an easy and fast assay to determine biochemical properties and behavior patterns in the heterologously produced LPMOs. Furthermore, both enzymes show a highly reactive and more stable activity at 50 °C for cellulose degradation. *Ps*LPMOA gave doubly oxidized compounds corresponding to C4 oxidation (4-keto or gem-diol sugar) and C1 oxidation (aldonic acid), while *Ps*LPMOB showed C4-oxidized cello-oligosaccharides products. This study sheds light on the molecular and structural basis for halotolerance in enzymes, raising prospects for engineering this characteristic to suit industrial biofuel production needs. Using salt-responsive *Ps*LPMOA could allow the use of sea water in biorefineries.

## Methods

### Chemicals, gene sequence, and microorganisms

All chemicals were of the highest purity grade available and were purchased from Sigma-Aldrich (Saint-Quentin-Fallavier, France) unless stated otherwise. Amplex Red (10-acetyl-3,7-dihydroxyphenoxazine) was purchased from VWR (Fontenay-sous-Bois, France). Restriction endonucleases and T4 DNA ligase were obtained from Fermentas-Thermo Fisher Scientific (Illkirch, France) and used as recommended by the manufacturer. Putative *lpmo* nucleotide sequences were identified in a transcriptome analysis of the mangrove fungus *Pestalotiopsis* sp. NCi6 [30]. Two of these sequences, *pslpmoa* (Genebank ID KR825270) and *pslpmob* (Genebank ID KR825269), were selected for expression. The cDNAs of *pslpmoa* and *pslpmob* were artificially synthesized with their native signal sequences and were codon-optimized for expression in *P. pastoris*. *N. crassa* CDH (IIA and IIB) was produced and purified as previously reported [24].

### Cloning and protein production of *Pestalotiopsis* sp. NCi6 LPMOs

The synthetic *pslpmoa* and *pslpmob* genes were digested with the restriction enzymes *Xho*I and *Xba*I and ligated into the equally treated vector pPICZα A (Invitrogen, Cergy-Pontoise, France) using the Rapid DNA Ligation Kit (Fermentas). The procedures resulted in plasmids carrying genes encoding proteins with their native signal sequences cloned under the control of the methanol-inducible AOX1 promoter. C-terminal 6× histidine tags for purification were included. The plasmids were linearized with the restriction enzyme *Sac*I and transformed into electro-competent *P. pastoris* cells. Transformants were grown on YPD plates (10 g L^−1^ yeast extract, 20 g L^−1^ peptone, 10 g L^−1^ glucose, 15 g L^−1^ agar) containing 100 mg L^−1^ Zeocin. Zeocin-resistant *P. pastoris* transformants were then screened for protein production by the platform method [39]. The best-producing transformant was grown in 1 L of BMGY containing 1 mL L^−1^ of PTM_4_ salts (2 g L^−1^ CuSO_4_·5H_2_O; 3 g L^−1^ MnSO_4_·H_2_O; 0.2 g L^−1^ Na_2_MoO_4_·2H_2_O; 0.02 g L^−1^ H_3_BO_3_; 0.5 g L^−1^ CaSO_4_·2H_2_O; 0.5 g L^−1^ CaCl_2_; 12.5 g L^−1^ ZnSO_4_·7H_2_O; 22 g L^−1^ FeSO_4_·7H_2_O; biotin 0.2 g L^−1^; conc. H_2_SO_4_ 1 mL L^−1^) in shaken flasks at 30 °C in an orbital shaker (200 rpm) for 16 h to an OD_600_ of 2–6. Gene expression was induced by transferring cells into 200 mL BMMY containing 1 mL L^−1^ of PTM4 salts at 25 °C in an orbital shaker (200 rpm) for another 3 days. The medium was supplemented with fresh 3 % (v/v) methanol every day.

### Recombinant production of LPMOs in a bioreactor and protein purification

The batch phase was performed in a 2-L bioreactor (MBR Electronics, Wald, Switzerland) with a starting volume of 1.2 L at 30 °C with the agitation set to 600 rpm. pH was kept constant at 5.0 using ammonium hydroxide (28 % v/v). Total O_2_ flow was between 0.1 and 0.2 vvm throughout the batch phase. The fermentations were started by adding 100 mL preculture grown overnight on YPD medium in 1-L baffled shaking flasks at 150 rpm and 30 °C. The cultivations were performed according to Invitrogen’s *Pichia* fermentation process guidelines with some alterations. After depletion of glycerol in the batch medium, the fed-batch phase was started with a constant feed of 36 mL h^−1^ 50 % glycerol containing 12 mL L^−1^ PTM1 trace salts overnight to increase biomass. For induction, the feed was switched to 100 % methanol containing 12 mL L^−1^ PTM1 trace salts at an initial feed rate of 12 mL h^−1^ until the culture was fully adapted to methanol. After induction, cultivation temperature was reduced to 25 °C. Dissolved oxygen in the bioreactor was held at 20 %. Samples were taken twice a day at 6 h intervals to measure wet biomass, protein concentration, and OD_600_.

The fermentation broths were centrifuged at 6000×*g* for 30 min and 4 °C, and sodium hydroxide (2 M) was used to adjust the pH to 7.8. An additional centrifugation step was performed before loading the clear supernatant onto a 64-mL Nickel-chelated ion metal affinity column (GE Healthcare Life Sciences, Marlborough, MA) equilibrated with 50 mM Tris–HCl, pH 7.8 containing 150 mM NaCl and 10 mM imidazole. (His)_6_-tagged enzymes were eluted within a linear gradient from 0 to 50 % of 500 mM imidazole within 3 column volumes, and 10-mL fractions were collected. Fractions containing recombinant enzymes were pooled, concentrated, and dialyzed against 50 mM potassium phosphate buffer pH 6.0 and stored at 4 °C. Bulk fractions were concentrated by diafiltration using a Vivaflow 50 cross-flow module (Sartorius, Göttingen, Germany) with a polyether-sulfone membrane and a cut-off of 10 kDa. Subsequently, fractions were further concentrated by Amicon centrifugation tubes (Millipore, Guyancourt, France) (cut-off 10 kDa, 3200×*g* for 15 min at 4 °C).

### Protein analysis and Western blotting

Protein contents of crude preparations or partially purified fractions were determined by the Bradford dye-binding method using a pre-fabricated assay (Biorad Laboratories, Marnes-la-Coquette, France) and BSA as calibration standard. Protein concentrations of purified enzymes were measured based on their molar absorption coefficients calculated from the mature amino acid sequence using ProtParam (http://www.web.expasy.org/protparam/). The molar absorption coefficients of *Ps*LPMOA and *Ps*LPMOB at 280 nm are 30.62 and 33.14 mM^−1^ cm^−1^, respectively. Concentrations of *Nc*CDHIIA and *Nc*CDHIIB were determined at 420 nm (103 and 87 mM^−1^ cm^−1^, respectively). Protein purification steps and fermentation progress were tracked by sodium dodecyl sulfate–polyacrylamide gel electrophoresis (SDS-PAGE) (Bio-Rad Laboratories). Protein bands were visualized with Coomassie Brilliant Blue R-250. The molecular mass under denaturing conditions was determined with reference standard proteins (PageRuler Prestained Protein Ladder, Thermo Fisher Scientific). All procedures followed the manufacturer’s recommendations (Bio-Rad Laboratories). Electrophoresed proteins were electroblotted onto polyvinylidene difluoride membranes following the manufacturer’s procedure (iBlot, Life Technologies, Saint-Aubin, France) [40].

### LPMO activity based on H_2_O_2_ analysis

The futile oxygen-reducing activity of LPMOs in the absence of a cellulosic substrate was used as proxy for enzyme activity. The assay is based on the peroxidase-dependent conversion of Amplex Red to the fluorescent resorufin and allows the time-resolved quantification of peroxide. The reaction stoichiometry (H_2_O_2_:Resorufin) is 1:1. [26]. Assays were performed in 96-well black polystyrene plates (total volume 200 µL) using a Perkin Elmer EnSpire Multimode plate reader (Perkin Elmer, Waltham, MA). An experimentally determined excitation wavelength of 569 nm and an emission wavelength of 585 nm were used. All reactions were performed in 100 mM sodium phosphate buffer, pH 5.5, containing 50 μM Amplex Red, 7.1 U mL^−1^ horseradish peroxidase with either 30 µM ascorbate, or 0.4 µM *Nc*CDH at 22 °C. We used 500 µM of cellobiose as an electron donor for *Nc*CDH. In reference experiments without LPMO, the background signal (H_2_O_2_ production by *Nc*CDH) was measured and subtracted from the assays. To determine the pH profile for *Ps*LPMOs, the signal intensity of resorufin fluorescence at different pH-values (pH 4.0–8.0) was determined by adding assorted concentrations of H_2_O_2_ (0.1–3.0 µM) to the assay for 20 min. A linear relation of fluorescence signals and H_2_O_2_ concentrations was found at all pH-values. Slopes were used to calculate the pH-dependent enzyme factors. LPMO activity was defined as one µmol H_2_O_2_ generated per minute under the defined assay conditions. LPMOs alone or in combination with cellobiose showed no signal that exceeded the signal of Amplex Red alone over the investigated timespan. Time-dependent degradation curves for the *Ps*LPMOs were measured by incubating sample aliquots of 70 µL for up to 1500 min at varying temperatures. Samples were sequentially placed in a thermomixer (±1 °C) and harvested at the same time to minimize measurement errors. Furthermore, sea salt effects on the activity of *Ps*LPMOs were tested using the standard Amplex Red assay and addition of 3.5 % (w/v) sea salt to the buffer. All measurements were performed at least in triplicate.

### N-terminal amino acid sequence determination and protein deglycosylation

The N-terminal amino acid sequences of purified *Ps*LPMOA and *Ps*LPMOB were determined according to Edman degradation. Samples were taken from *Ps*LPMOs electroblotted onto a polyvinylidene difluoride membrane (iBlot, Life Technologies). Analyses were carried out on an Applied Biosystems 470A by the proteomics platform at the Institut de Microbiologie de la Méditerranée, CNRS-Aix-Marsille University, Marseille, France. Recombinant *Ps*LPMOs were deglycosylated using N-glycosidase F (PNGase F) as per manufacturer’s procedure (New England Biolabs, Évry, France) under denaturing conditions. Glycosylation sequences were predicted using NetNGlyc 4.0 (http://www.cbs.dtu.dk/services/NetNGlyc/) and NetOGlyc 3.1 (http://www.cbs.dtu.dk/services/NetOGlyc/).

### Cellulolytic activity and regioselectivity of *Ps*LPMOs

Phosphoric acid swollen cellulose (PASC, prepared from Avicel as described in [41]) was incubated with *Ps*LPMOs and analyzed for cellulose-oligosaccharides (Megazyme, Wicklow, Ireland). All cleavage assays (300 μL liquid volume) contained 5 μM *Ps*LPMOs, 1 mM ascorbic acid and 0.1 % (w/v) PASC in 50 mM sodium acetate buffer, pH 5.5. Control experiments were performed without ascorbic acid in the presence of *Ps*LPMOs. All experiments were performed in 2-mL tubes incubated in a thermomixer (Eppendorf, Montesson, France) at 50 °C and 850 rpm for 24 or 72 h. All samples were boiled at 100 °C for 10 min to stop the enzymatic reaction and then centrifuged at 16,000×*g* for 15 min at 4 °C to separate the soluble fraction from the remaining insoluble fraction before product analysis. *Nc*CDHIIA was used in the subsequent reactions to determine the regioselectivity of *Ps*LPMOs. *Nc*CDHIIA was used at a concentration of 1.2 U mL^−1^ along with 400 µM cellobiose as electron donor and incubated for 22 h with the soluble fraction obtained after action of *Ps*LPMOs. The reactions were carried out as described above. To determine effect of sea salt on activity of *Ps*LPMOs, experiments were performed as described above with addition of 0.0–6.0 % (w/v) of sea salt into reaction mixtures. Blank controls were performed under the same conditions without ascorbic acid or *Nc*CDHIIA, and *Ps*LPMOs. All assays were performed as triplicate-independent experiments.

### Analysis of oxidized and non-oxidized cello-oligosaccharides

Mono- and oligosaccharides as well as their corresponding aldonic acid forms generated after PASC cleavage were analyzed by HPAEC as described previously [21, 37]. Non-oxidized cello-oligosaccharides were used as standards. Corresponding oxidized standards were produced from non-oxidized cello-oligosaccharides by CDH treatment as described in [21].

### Sequence analysis and homology models of *Ps*LPMOs

A sequence alignment of *Ps*LPMOs with biochemically characterized LPMOs from other species was performed using the Clustal Omega multiple sequence alignment program (http://www.ebi.ac.uk/Tools/msa/clustalo/). Structural homology models for all *Ps*LPMOs were generated using the SWISS-MODEL server (http://www.swissmodel.expasy.org/) [42]. Quality assessments including Ramachandran plots for all models were performed with MolProbity (http://www.molprobity.biochem.duke.edu/). Structures were visualized using the PyMOL molecular graphics system, version 1.4 (Schrödinger, New York, NY, USA).

## Additional file

10.1186/s13068-016-0520-3 Production, purification and characterization of lytic polysaccharide monooxygenases secreted by *Pestalotiopsis* sp. NCi6.

## Abbreviations

LPMOs: lytic polysaccharide monooxygenases
AA: auxiliary activities
CDH: cellobiose dehydrogenase
PASC: phosphoric acid swollen cellulose
HPAEC: high-performance anion-exchange chromatography
