# Supplementary material for: Salt-responsive lytic polysaccharide monooxygenases from the mangrove fungus Pestalotiopsis sp. NCi6
Source: Biotechnol Biofuels. 2016 May 20;9:108. doi: 10.1186/s13068-016-0520-3 (PMC4875668; doi:10.1186/s13068-016-0520-3)
Supplement: Supplementary file 1 — 10.1186/s13068-016-0520-3 Production, purification and characterization of lytic polysaccharide monooxygenases secreted by Pestalotiopsis sp. NCi6. [file 13068_2016_520_MOESM1_ESM.doc]

**Supplementary data**

Salt-responsive lytic polysaccharide monooxygenases from the mangrove fungus *Pestalotiopsis* sp. Nci6

Ilabahen Patel^1,2*^, Daniel Kracher^3^, Su Ma3, Sona Garajova^1,2^, Mireille Haon^1,2^, Craig B. Faulds^1,2^, Jean-Guy Berrin^1,2^, Roland Ludwig^3^, Eric Record^1,2*^


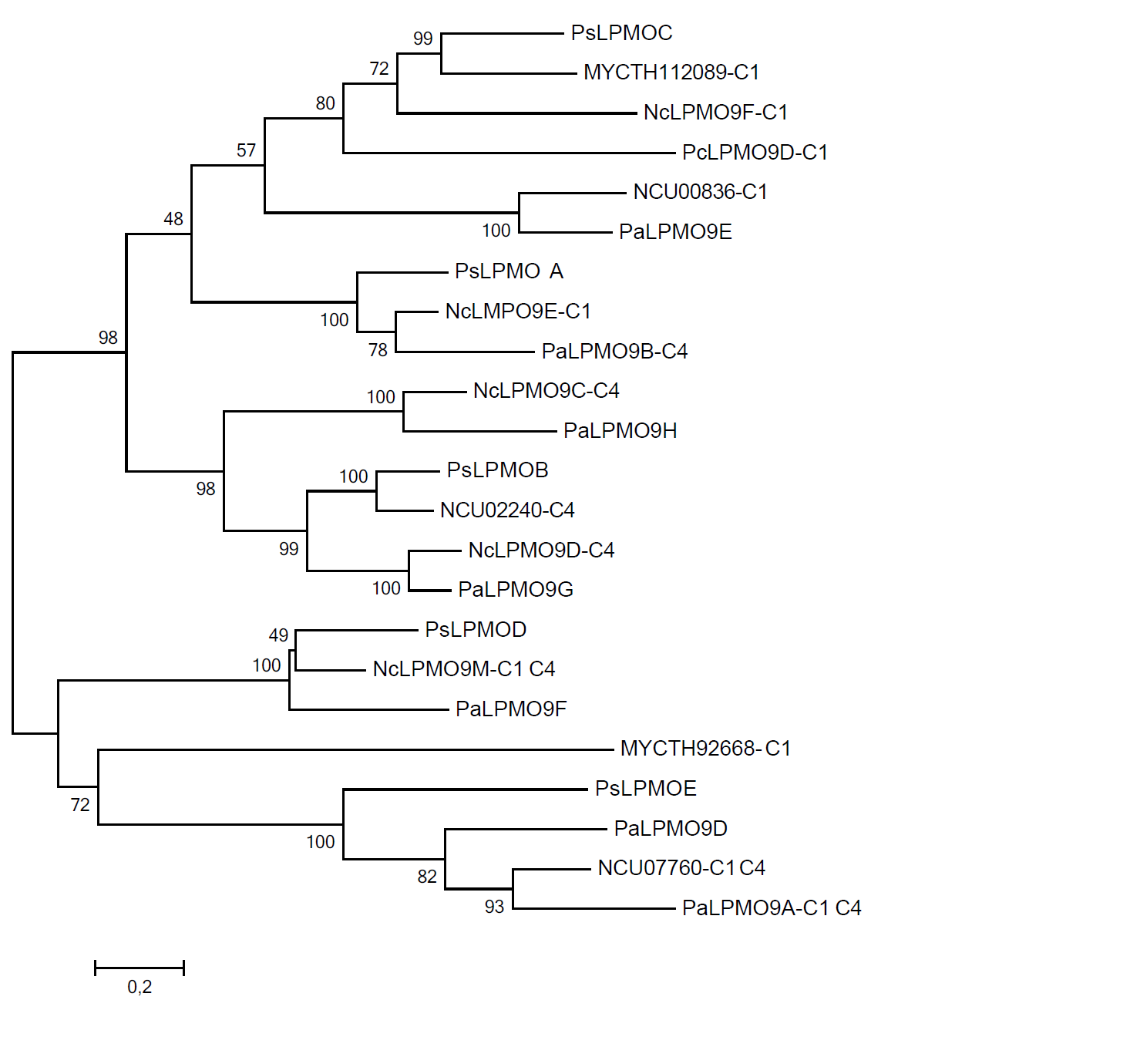


PsLPMOC (Genebank ID KR825268)

PsLPMOA (Genebank ID KR825270)

PsLPMOB (Genebank ID KR825269)

PsLPMOD (Genebank ID KR825271)

PsLPMOE (Genebank ID KR825272)

**Figure S1** Phylogenetic tree of *Ps*LPMOs and previously characterized LPMOs.


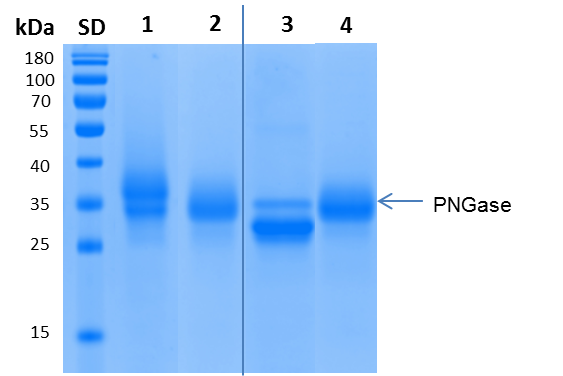


**Figure S2** SDS-PAGE analysis of purified LPMOs and deglycosylated *Ps*LPMOs. SD, standard molecular marker; Lane 1, purified *Ps*LPMOA; Lane 2, purified *Ps*LPMOB; Lane 3, *Ps*LPMOA treated with PNGase; Lane 4, *Ps*LPMOB treated with PNGase.


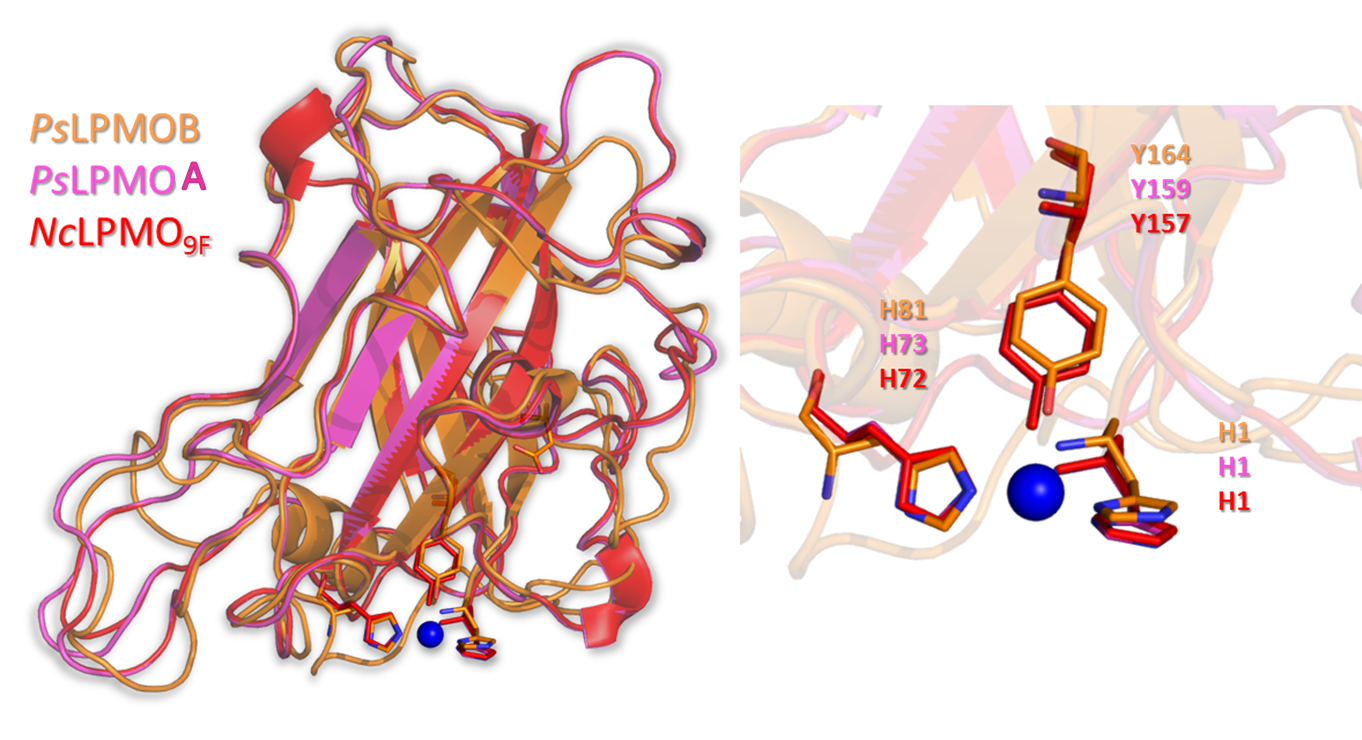


**Figure S3**. Structural overlay of *Ps*LPMOA and *Ps*LPMOB homology models with the crystal structure of *Nc*LPMO_9F_ (pdb: 4QI8;[34]). Active site geometries are shown in the inset.

**Table S1** Charge properties of various LPMOs from different origins. Theoretical isoelectric points were calculated with protparam (<http://web.expasy.org/protparam/>). *Ps*, *Pestalotiopsis* sp. NCi6; *Nc*, *Neurospora crassa*; *Hj*, *Hypocrea jecorina*; *Pc*, *Phanerochaete chrysosporium*; *Tt*, *T. terrestris*; *Ta*, *Thermoascus auranthiacus*; *Mt*, *Myriococcum thermophilum*; *Pa*, *Podospora anserina*.

| **LPMO** | **UniProt** | **Asp + Glu** | **Arg + Lys** | **Ratio (D+E)/(R+K)** | **pI calculated** | **pI measured** |
| --- | --- | --- | --- | --- | --- | --- |
| ***Ps*LPMOA** | XP_007830945 | **24** | **5** | **4.8** | **4.29** | **4.2-4.7** |
| ***Ps*LPMOB** | XP_007831549 | **26** | **6** | **4.3** | **3.95** | **4.2-4.7** |
| *Nc*PMO2 | 4EIR | 15 | 14 | 1.1 | 6.46 |  |
| *Nc*LPMO9F | 4QI8 | 10 | 13 | 0.8 | 8.65 |  |
| *Nc*LPMO9C | 4D7U | 15 | 13 | 1.2 | 6.19 |  |
| *Nc*LPMO2240 | XP_331016.1 | 18 | 11 | 1.6 | 4.82 |  |
| *Nc*LPMO9M | 4EIS | 16 | 14 | 1.1 | 6.3 |  |
| *Nc*NCU07760 | XP_328466.1 | 27 | 16 | 1.7 | 4.89 |  |
| *Nc*NCU00836 | XP_325016.1 | 15 | 10 | 1.5 | 5.37 |  |
| *Nc*LMPO9E | XP_330877.1 | 19 | 16 | 1.2 | 6.04 |  |
| *Hj*LPMO | 2VTC | 11 | 11 | 1 | 7.15 |  |
| *Pc*LPMO | 4B5Q | 19 | 8 | 2.4 | 4.54 |  |
| *Tt*LPMO | 3EJA | 15 | 10 | 1.5 | 5.14 |  |
| *Ta*LPMO | 3ZUD | 19 | 7 | 2.7 | 4.59 |  |
| *Mt*MYCTH112089 | AEO60271.1 | 16 | 8 | 2 | 5.02 |  |
| *Mt*MYCTH92668 | AEO56665.1 | 21 | 13 | 1.6 | 5.06 |  |
| *Pa*LPMO9B | CDP31846.1 | 18 | 16 | 1.1 | 6.38 |  |
| *Pa*LPMO9A | CDP25655.1 | 15 | 11 | 1.4 | 6.02 |  |
| *Pa*LPMO9D | CDP28479.1 | 19 | 14 | 1.4 | 5.74 |  |
| *Pa*LPMO9E | CDP23998.1 | 10 | 10 | 1 | 7.1 |  |
| *Pa*LPMO9F | CDP31230.1 | 14 | 13 | 1.1 | 6.69 |  |
| *Pa*LPMO9G | CDP25137.1 | 12 | 13 | 0.9 | 7.85 |  |
| *Pa*LPMO9H | CDP27830.1 | 15 | 13 | 1.2 | 6.52 |  |


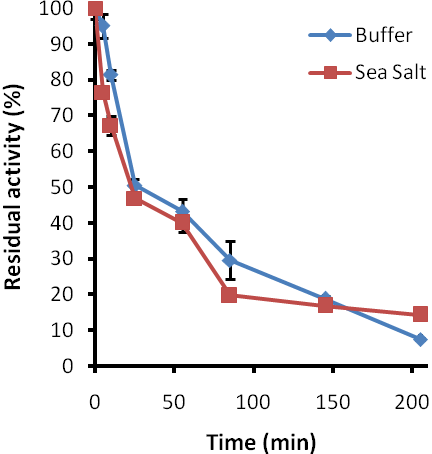

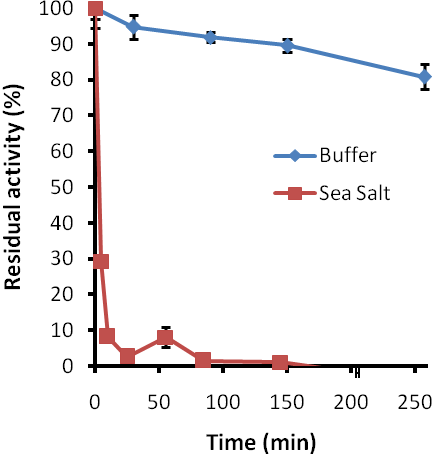


***Ps*LPMOB**

***Ps*LPMOA**

**Figure S4**. Effect of sea salt on the temperature stability of *Ps*LPMOs. Activity was measured using the Amplex Red assay as described in Materials and Methods. Sea salt was added to a final concentration of 3.5% (w/v). Error bars are the mean of three replicates.


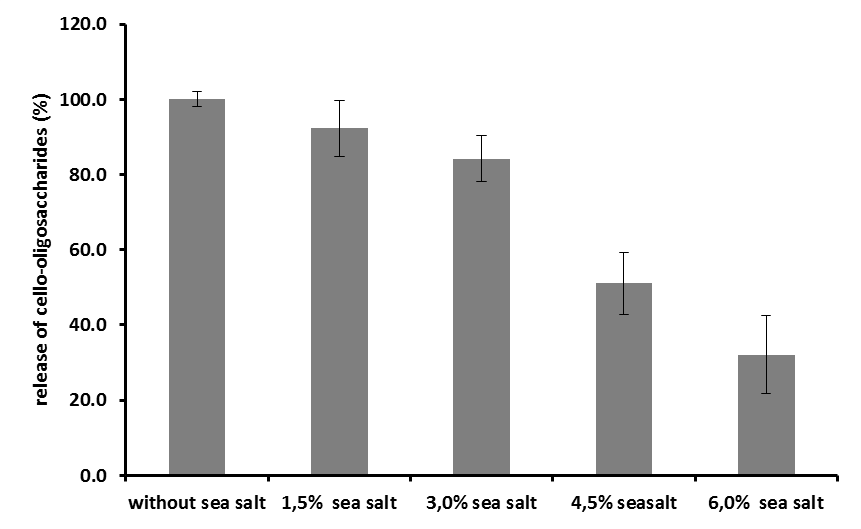


**Figure S5.** Quantification of the cello-oligosaccharides (oxidized and non-oxidized oligosaccharides) released by action of *Ps*LPMOA in presence of different concentrations of sea salt by HPAEC analysis. The concentration of each oligosaccharides was determined by integration of the peak area for each sample and comparison with a standard curve. Error bars are the mean of three replicates.
